# Suicide among university students: prevalence, risks and protective factors

**DOI:** 10.1080/21642850.2020.1766978

**Published:** 2020-06-05

**Authors:** Frances Emily Owusu-Ansah, Akua Afriyie Addae, Bernice Ofosuhene Peasah, Kwaku Oppong Asante, Joseph Osafo

**Affiliations:** a Counselling Centre, Kwame Nkrumah University of Science and Technology, Kumasi, Ghana; b Department of Behavioural Sciences, Kwame Nkrumah University of Science and Technology, Kumasi, Ghana; c Department of Psychology, University of Ghana, Accra, Ghana; d Department of Psychology, University of the Free State, Bloemfontein, South Africa

**Keywords:** Suicide, risk, protective factors, university students, Ghana

## Abstract

**Background:** Research evidence on suicide in Ghana so far has focused mostly on suicide in the adult population and less work on the younger population such as university students and other vulnerable groups such as children, youth and the aged.

**Aims:** This study was conducted to determine lifetime and current prevalence of suicidal ideation or attempts and identify the associated risks and protective factors among university students in Ghana.

**Methods:** Using a cross-sectional design, 1003 university students (507 males and 496 females) with a mean age of 20.5 years (SD = 5.95) were administered questionnaires that measured suicide, psychological distress, self-esteem and subjective wellbeing.

**Results:** We found the following prevalence rates of suicidal behaviours: ideations 15.2%, attempted 6.3%, death wishes 24.3% and suicidal plan 6.8%. Psychological distress was a risk factor for both suicidal ideation and suicidal attempt. Subjective wellbeing was protective of suicide attempt while self-esteem was protective of suicidal ideation.

**Conclusions:** These findings underscore the need for school-based mental health-promoting programmes that enhance young people’s self-esteem, reduce psychological distress and boost subjective wellbeing.

## Introduction

Suicide is a public health concern of great importance given the enduring devastating effects it has on families, friends and communities. Annually, it claims over 800,000 lives (WHO, 2014) and many of these are young people within the 15–29 age bracket (Nock et al., 2013; WHO, 2012); a situation with grave economic implications for our world today (Kinyanda, Hoskins, Nakku, Nawaz, & Patel, 2012). Hence, the World Health Organization’s Gap Action Programme of 2008 included suicide as one of the priorities for serious attention and action.

Country-specific empirically validated interventions must be informed by evidence-based research. Yet compared to the depth of knowledge on suicide, risk and prevention, in the western world (Bridge, Goldstein, & Brent, 2006; Fontenella et al., 2015; Van Geel, Vedder, & Tanilon, 2014), there is relatively a paucity of suicide research in Africa (Adinkrah, 2011; Osafo, Akotia, & Boakye, 2016); although in Ghana some efforts have been made in the last decade that shed light on the phenomenon (Adinkrah, 2011; Osafo, Hjelmeland, Akotia, & Knizek, 2011).

Some progress notwithstanding, the research evidence on suicide in Ghana so far has focused mostly on suicide in the adult population (Adinkrah, 2012; Akotia, Knizek, Kinyanda, & Hjelmeland, 2014; Asare-Doku, Osafo, & Akotia, 2017; Osafo et al., 2016; Osafo, Akotia, Andoh-Arthur, & Quarshie, 2015; Sefa-Dedeh & Canetto, 1992) and less work on the younger population such as university students and other vulnerable groups such as children, youth and the aged (Eshun, 1999, 2003; Nanewortor, 2011).

Information on completed suicides is difficult to obtain. Hence, the increased research concentration on delineating risk factors for suicidal ideation and attempts (Osman et al., 1998). Though reliable and official statistics on suicide among university students in Ghana may be lacking, some studies provide insights about risks for a better understanding of the occurrence among this group. Quarshie et al. (2019) provided germinal insights on suicide among nursing and midwifery students. Other studies, while not specifically focusing on suicide behaviour, found alarming prevalence rates of depression (mild, moderate and severe), inadequate social support, substance abuse, self-stigma and traumatic life events among university students (Andoh-Arthur, Oppong Asante, & Osafo, 2015; Oppong Asante & Andoh-Arthur, 2015). Anxiety, loneliness, poor self-esteem, anger management problems (Peltzer, Kleintjes, Wyk, Thompson, & Mashego, 2008) and academic-related challenges that ultimately impede subjective wellbeing have also been found (Oppong Asante, Kugbey, Osafo, Quarshie, & Sarfo, 2017) and potentially form the genesis of psychological distress and poor mental health as contributors to suicidal behaviour.

In-country findings on risk indicators for suicide as well as buffers are consistent with studies conducted elsewhere around the world. Some evidence from adult sample studies on suicide in Ghana have shown that predisposing risk factors include depression, impulsivity, poverty, poor neighbourhood, lack of parental warmth and/or abuse and family conflict (Osafo et al., 2016). Others have found strong positive connections between levels of depression (Bantjes, Kagee, McGowan, & Steel, 2016; Korb & Plattner, 2014; Wang et al., 2014), anxiety (Choi et al., 2011), substance use and risk of suicide ideation among university students (Ashrafioun, Bonar, & Conner, 2016; De Luca, Franklin, Yueqi, Johnson, & Brownson, 2016; Yen, Liu, Yang, & Hu, 2015). Positive correlations between impulsivity, parental neglect (Swahn, Palmier, Kasirye, & Yao, 2012), aggression, lack of satisfaction with an aspect of their lives (e.g. their major of study) and suicidal behaviour have been reported (Wang et al., 2014).

Evidence on protective factors include good social support, self-esteem, maintenance of healthy and positive relationships (Sarchiapone, Mandelli, Iosue, Andrisano, & Roy, 2011; WHO, 2014). Parental connectedness and understanding (Borowsky, Taliaferro, & McMorris, 2013; Oppong Asante et al., 2017), and respect for the privacy of students have protective effects. Religious affiliation and beliefs have also been found to positively reduce risk of suicidal behaviour among at-risk youth (Baetz & Bowen, 2011; Griffin-Fennell & Williams, 2006).

Statistics from census report in Ghana (Ghana Statistical Service, GSS, 2013) shows that over 30% of recorded deaths categorized under *deaths by accident, violence, homicide or suicide*, occurred among the youth within the age range of pre-university and university populace. Existing evidence suggests that suicide among youth is becoming a daily reality in Ghana (Quarshie, Osafo, Akotia, & Peprah, 2015). Yet the extent to which reported risk factors reflect and affect suicidality in university students is yet to be examined. In Ghana there is still much to be understood in order to effectively design context-specific interventions.

Suicidal ideation among university students is undoubtedly a multifaceted phenomenon with globally acknowledged negative and deleterious effects on families, friends and even the socio-economic development of countries within which it occurs. Among young people, especially university students, suicide is the second leading cause of death besides self-inflicted injuries, making this population an at-risk group (Nock et al., 2013; Taliaferro et al., 2009a, 2009b; WHO, 2012). Therefore, importance of research for greater understanding of this menace cannot be overemphasized.

Theoretically, the present study examines suicide from a human ecological model perspective in a bid to understand the occurrence of suicide among university students (Bronfenbrenner, 1977, 1979; Hawton & van Heeringen, 2002; van Orden et al., 2010). Just as suicide risks, as well as buffers, may be complex to unravel the model offers a conceptualization of suicide from a multifaceted approach integrating the individual, social and familial factors that may be associated with suicide occurrence (e.g. Ayyash-Abdo, 2002; Henry, Stephenson, Hanson, & Hargett, 1993); and thus afford a broader environmental context interpretation for a better understanding and elucidation of risk factors.

Scrutiny of the research on suicide in Ghana shows a gap as far as information on suicide among university students is concerned. This study aims to address the gap by focusing the research on university students and so add to existing information for the design of effective interventions for this at-risk group. Therefore, the twofold-specific objectives of the study were to determine lifetime and current prevalence of suicidal ideation or attempts; and to identify associated risks and protective factors. Based on existing literature, the study predicts that students who report higher levels of psychological distress but have lower levels of self-esteem and subjective wellbeing would be more likely to exhibit suicidal behaviours (herein ideations and/or attempts). In other words, a negative relationship is expected between suicidal ideations or attempts and these variables. Likewise, students with poorer self-esteem are expected to be less satisfied with their lives and be more psychologically distressed. Since greater subjective wellbeing has been associated with greater perceptions of control over important life choices (Owusu-Ansah, 2008; Owusu-Ansah, Agyei-Baffour, & Edusei, 2012), a greater subjective wellbeing is also expected to protect against suicidal ideations and/or attempts.

## Methods

### Study design and participants

The study was a cross-sectional design as it aimed to establish a possible relationship among various variables. This method of study allows the researchers to establish and measure relationships among study variables. This design was used because it entails surveys or other pre-structured means that helped to obtain a common dataset on some pre-selected variables. One thousand and three (1003) students purposively selected participated in the study. Participants were recruited through the *Know Your Status’* screening exercise: a screening exercise annually organized by the KNUST Counselling Centre (KCC) during which students voluntarily test for HIV/AIDS and Hepatitis B status, blood group and undergo breast examinations. Participation in the study was limited to students who attended the KCC programme. The sample size was determined a priori using GPower software version 3.1 (Faul, Erdfelder, Lang, & Buchner, 2007). With a power value of 0.80, a significant level of 0.10, and a minimum effect size of 0.10, the minimum sample size was calculated to be 448. Thus, a sample size of 1003 was found to be adequate.

### Setting

The study was conducted among students of the Kwame Nkrumah University of Science and Technology (KNUST). The KNUST is one of the public and government-funded universities in Ghana located in Kumasi, in the Ashanti Region. It has a student population of over 40,000 and staff strength of approximately 3610. The university comprises of six colleges, namely College of Agriculture and Natural Resources (CANR), College of Art and Built Environment (CABE), College of Engineering (CoE), College of Humanities and Social Sciences (CoHSS), College of Health Sciences (CHS) and College of Science (CoS).

### Procedure

Participants were provided adequate information about the study and had their questions about the study answered. Having understood what was required of them, those we voluntarily choose to participate were given questionnaires to complete. All key ethical principles of informed consent, voluntary participation and confidentiality were strictly adhered to throughout the study. The study protocol was reviewed and approved by the Committee on Human Research, Publication and Ethics (CHRPE/RC/253/17) of the School of Medicine and Dentistry (SMD), Kwame Nkrumah University of Science and Technology.

### Measures

#### Demographics

Demographic characteristics assessed included sex, marital status, religion, year of study, college, residence and nationality.

##### Suicide-related items

*Lifetime suicidal attempt*, for instance, was measured by asking ‘have you personally attempted to end your life?’ to which the participants answered ‘yes’ or ‘no’. Current suicidal attempt was elicited with the question ‘If yes, how many times has this happened in the last 12months?’ *Lifetime suicidal ideation* was measured by asking participants ‘Have you ever thought about ending your own life? *Lifetime death wishes* was measure by asking participants ‘Is there a time in your life when you wished were dead?’ *Lifetime suicide plan* was measured by asking participants ‘Have you ever had a clear plan for killing yourself’.

##### Self-esteem

Participants’ self-esteem was measured with the Rosenberg Self-Esteem Scale (RSE). This is a 10-item questionnaire that asks respondents to indicate how they feel about themselves. Some of the items include ‘On the whole, I am satisfied with myself’, ‘At times I think I am no good at all’. Participants indicate the extent to which they agree with these statements. Responses are anchored on a four-point Likert scale ranging from strongly agree (1) to strongly disagree (4). Five of the items are negatively worded while five are positively worded. Negatively worded items are reversed scored such that higher scores indicate more positive self-esteem. An individual’s score is obtained by summing his/her responses. Scores ranged from 10 to 40. The validity of the Rosenberg Self-Esteem Scale is well established within the Ghanaian context (Nyarko, 2017).

##### Subjective wellbeing

The Ryff’s Psychological Wellbeing (PWB) Scale an 18-item questionnaire was used to measure psychological wellbeing. Some of the items are ‘When I look at the story of my life, I am pleased about how things have turned out’ and ‘I don’t have a good sense of what it is I am trying to accomplish in life’*.* Responses are scored on a five-point Likert scale ranging from strongly disagree (1) to strongly agree (5). Negatively worded items are reversed scored and an individual’s score is obtained by summing responses. Scores range from 18 to 90 with higher scores indicating higher or better subjective wellbeing and satisfaction with one’s life. The PWB Scale has shown good psychometric properties in adult samples of all ages and backgrounds (Curhan et al., 2014; Ryff & Keyes, 1995) as well as college students (Gloria, Castellanos, Scull, & Villegas, 2009; Ryff, Keyes, & Hughes, 2003).

##### Psychological distress

This variables was assessed using the Depression Anxiety Stress Scale (DASS-21) (Lovibond & Lovibond, 1995) The DASS-21 is a well-structured questionnaire consisting of three subscales of seven items each measuring depression, anxiety and stress. The depression subscale assessed the presence of self-blame, pessimism and loss of enjoyment. The anxiety subscale assessed the state of persistent apprehension and worry, accompanied by physical symptoms of sympathetic activation. The stress subscale assessed the state of over-arousal, tenseness and the inability to relax. The internal consistencies of the three subscales were .71, .79 and .81 for depression, stress and anxiety, respectively. Items on the DASS-21 are scored on a three-point Likert scale ranging from 0 = does not apply to me at all to 3 = applies to me very much or most of the time. The reliability of the DASS-21 has been established within the Ghanaian context (Arhin, Oppong Asante, Kugbey, & Oti-Boadi, 2019; Asante, 2012). Since, the DASS-21 distinguishes between depression, anxiety and stress as distinct manifestations of psychological distress, the total of the subscales were used in this study.

### Data analysis

The Statistical Package for Social Sciences (SPSS) version 24.00 was used to analyse the data. Descriptive statistics was used to describe the nature of the data through the generation of frequencies and percentages. Pearson’s correlation analysis was used to test the relationships among the study variables. As the outcomes variables were categorical in nature (i.e. suicide ideation and attempts), logistic regression analyses were conducted to determine the predictors of both suicidal ideations and attempts. The results from the logistic regression analyses are presented as odds ratio (OR) and 95% confidence interval (CI). All statistical tests were performed using a two-tailed examination, and a *p* value of 0.05 or less was considered statistically significant.

### Ethics statement

The study protocol was reviewed and approved by the Committee on Human Research, Publication and Ethics (CHRPE/RC/253/17) of the School of Medicine and Dentistry (SMD), Kwame Nkrumah University of Science and Technology.

## Results

### Demographic characteristics of the sample

The demographic characteristics of the sample are presented in Table 1. The sample included 1003 university students (507 males and 496 females) with a mean age of 20.53 years (SD = 5.95). The majority (86.4%) of the students were single and half of them (50.3%) were first-year students. Approximately 94% of the students were Christians, 5.8% were Muslim and the remaining 0.5% belonged to other religious affiliations. The same number of students (23%) were from both the Humanities and Health Sciences colleges, while 21.7% of the students were from the Physical Science College. The majority of the students were Ghanaians (97.9%) and residents on campus (74.9%).

**Table 1.** Demographic characteristics of the sample.

Variables	Frequency	Percentages
Gender		
Male	507	50.5%
Female	496	49.5%
Marital Status		
Single	864	86.4%
In a relationship	127	12.7%
Married	9	0.9%
Religion		
Christian	910	93.7%
Muslim	56	5.8%
Others	5	0.5%
Year of study		
First year	485	50.3%
Second year	142	14.7%
Third year	236	24.5%
Fourth year	86	8.9%
Postgraduates	15	1.6%
Colleges		
Arts and Built Environment	106	10.6%
Humanities and Social Sciences	229	23.0%
Health Sciences	229	23.0%
Agric and Natural Sciences	96	9.6%
Engineering	120	12.0%
Sciences	216	21.7%
Residence		
On campus	451	74.9%
Off campus	251	25.1%
Nationality		
Ghanaian	907	97.9%
International student	19	2.1%

#### Prevalence of suicide

The prevalence of current and lifetime prevalence of suicide among students are presented in Table 2. Lifetime prevalence of suicidal attempts among the participants was 6.3%. About 15.2% reported suicidal ideation and almost a quarter of the participants had had death wishes. Approximately 7% have had clear suicide plans in their lifetime. With regard to lifetime prevalence, 23.8% had attempted suicide more than once while 25.0% had suicidal ideation more than once in the past year. Approximately 19% reported that they had more than once made plans for suicide in the past year.

**Table 2.** Suicidal behaviour among participants.

Variables	Frequency	Percentages
Current prevalence		
Attempted suicide	63	6.3%
Suicide ideations	152	15.2%
Death wishes	241	24.3%
Suicidal plan	68	6.8%
Lifetime prevalence: Attempted suicide		
None	23	36.5%
Once	25	39.7%
More than once	15	23.8%
Lifetime prevalence: Suicide ideation		
None	52	34.2%
Once	62	40.8%
More than once	38	25.0%
Lifetime prevalence: Death wishes		
None	64	26.9%
Once	96	40.3%
More than once	78	32.8%
Lifetime prevalence: Suicide plan		
None	26	38.2%
Once	29	42.7%
More than once	13	19.1%

#### Factors associated with suicide ideation and suicide attempt

The results of the logistic regression analysis of the predictors of suicide ideation and attempt among students are presented in Table 3. Suicidal attempt was significantly predicted by psychological wellbeing and psychological distress. Positive subjective or psychological wellbeing buffers against suicidal attempt (AOR = 0.95; CI 0.95, 1.05) while excessive psychological distress increases the likelihood of attempting suicide (AOR = 1.07; CI 1.04, 1.09). Positive self-esteem served as a buffer against suicidal ideation (AOR = 1.05; CI 0.90, 0.99) and the presence of psychological distress increased the likelihood of having suicidal ideations (AOR = 1.07; CI 1.04, 1.09). In suicidal ideation, self-esteem and psychological distress matter; while in suicidal attempts, subjective wellbeing and psychological distress are the risk factors. In other words, self-esteem seems to play a significant role in suicidal ideation as subjective wellbeing is in suicidal attempt aside the influence of psychological distress in both instances.

**Table 3.** Predictors of suicide ideation and suicide attempt.

Variables	Suicide ideation		Suicide attempt	
	OR (95%CI)	*p* values	OR(95%CI)	*p* values
Sex (Female)	1.40 (0.97–2.03)	0.073	1.71 (0.95–3.06)	0.072
Self-esteem	0.94 (0.90–0.99)	0.010	0.98 (0.91–1.05)	0.551
Wellbeing	0.99 (0.97–1.02)	0.683	0.94 (0.91–0.98)	0.007
Depression	1.06 (0.99–1.14)	0.099	0.99 (0.90–1.11)	0.981
Anxiety	1.06 (0.99–1.14)	0.068	1.25 (1.14–1.38)	<0.001
Stress	1.04 (0.96–1.12)	0.356	0.96 (0.86–1.08)	0.488
Cox & Snell R^2^	0.07		0.07	
Nagelkerke R^2^	0.13		0.20	
Chi-square (*χ*^2^) test = 72.65; *p* < 0.001			74.98; *p* < 0.001	

## Discussion

This study was conducted to determine lifetime and current prevalence of suicidal ideation or attempts and identify the associated risks and protective factors among university students in Ghana. We found the following prevalence rates of suicidal behaviours: ideations 15.2%, attempted 6.3%, death wishes 24.3% and suicidal plan 6.8%. Psychological distress was a risk factor to both suicidal ideation and suicidal attempt. Subjective wellbeing was protective of suicide attempt while self-esteem was protective of suicidal ideation.

### Suicide prevalence rates

The current prevalence rates of suicidal behaviours: ideations 15.2%, attempted 6.3%, death wishes 24.3% and suicidal plan 6.8% are fairly consistent with a recent study of nursing students on suicide in Ghana (Quarshie, Cheataa-Plange, Annor, Asare-Doku, & Lartey, 2019) as well as secondary school students (Oppong Asante et al., 2017; Baiden et al., 2019). In 2008, Hjelmeland and colleagues reported that only 4% of Ghanaian college students compared to 7% Ugandans and 8% Norwegians reported that they might consider suicide under some circumstances. Relatively lower percentages (2% and 4%) of Ghanaian college students reported own suicidal acts last year and earlier in life respectively in the current study. Eshun (2003) indicated that the low scores on suicide ideation by Ghanaian college students, compared with their counterparts elsewhere, are influenced by religion and negative attitudes towards suicide. The scores on suicide, although comparable to the global situation of suicide thoughts and behaviours of college students (Mortier et al., 2018) are slightly lower compared to samples in South Africa (e.g. Bantjes et al., 2016; van Niekerk, Scribante, & Raubenheimer, 2012). These low scores, more than a decade ago, are changing.

### Psychological distress and self-esteem predicting suicidal ideation

Our findings showed that psychological distress predicted suicidal ideation among university students. The presence of psychological distress increased the likelihood of suicidal ideations, consistent with general findings of a strong link of suicide to psychological distress (Tanji, Tomata, Zhang, Otsuka, & Tsuji, 2018; Walker, McGee, & Druss, 2015). It was also observed that low self-esteem is associated with suicide. Self-esteem has been identified in the WHO (2014) report on suicide prevention as an important protective buffer against stressors and suicidal behaviours for young people. A strong association between self-esteem difficulties and suicide ideation among young adults has been documented (Mirsu-Paun, 2015). The present findings corroborate these earlier reports.

### Psychological distress and wellbeing predicting suicidal attempt

We expected higher suicide attempts among students with greater psychological distress and higher subjective wellbeing to protect against such behaviours. This prediction was confirmed. Psychological distress positively correlated with suicide attempt, but subjective wellbeing was inversely related. Consistent with our finding, some reports show that subjective wellbeing is related with low rates in suicide (Hsu, Chang, & Yip, 2019) and correlates negatively with suicide intention (Sisask, Värnik, Kolves, Konstabel, & Wasserman, 2008). In one study among students, results indicated that the effects of all types of bullying on suicidal ideation were mediated by subjective wellbeing (Lucas-Molinaa, Pérez-Albénizb, & Fonseca-Pedrerob, 2018). In an ecological study analysing the geography of suicide in New Zealand among young people, Snider (2011) reported a correlation between low levels of psychological wellbeing and increased suicidal behaviours and self-harm for both males and females. Other studies have consistently shown that subjective wellbeing is generally protective of young people’s mental health in Eastern Europe (Helliwell, 2007; Lim, Cappa, & Patton, 2017); and in this study, protective of suicidality, in particular. Thus, what we have found in the present study reflects the critical role of subjective wellbeing in the mental health of Ghanaian university students.

### Implications for interventions

These findings have implications for scaling up mental health services in this population. Frist, mental health in Ghana is already underfunded and in many universities policies to improve the mental health of students are barely existent. As a country, Ghana has no clearly delineated policies for improving adolescent and young adults’ mental health. A major implication of this study is a clarion call to intensity efforts to promote mental health in universities in Ghana. For example, in Australia, the health-promoting schools approach model has been adopted (Wallace, Holloway, Woods, Malloy, & Rose, 2011) as recommended by the WHO to develop students’ social, emotional and behavioural competencies; identify at-risk students who might have such problems, treat and prevent further disorders among such individuals. Such school-based mental health-promoting approaches could pay attention to building young people’s self-esteem, reduce their psychological distress and boost subjective wellbeing.

Secondly as recommended in a recent study which examined suicidal behaviour among nursing trainees (Quarshie et al., 2019), the importance of periodic screening of students and the strengthening of onsite mental health counselling services for them is equally important. Since reports indicate only a moderate level of psychological help-seeking behaviours among university students in Ghana (Andoh-Arthur et al., 2015), mental health services for this population may not be successful if presented in the traditional waiting, reactive mode but rather in the seeking mode (Maseko, Maunganidze, Mambende, & Maphosa, 2017; Orford, 2008). That is, the practitioner need not wait for individuals to initiate contact, but rather makes efforts to bring services to clients, understand presenting problems and develop preventive intervention response programmes.

### Limitations of the study

This study is not without some limitations. First, there is a cultural revulsion against suicide (Adinkrah, 2011, 2012; Osafo, 2016) and thus hampers accurate reporting on such behaviours. The current prevalence rates might simply be a tip of the iceberg and may not reflect the true state of suicidal behaviour among university students in Ghana. Secondly, causal inferences cannot be made from cross-sectional data, though the direction and strength of associations give us clues. Thirdly, caution should be exercised in the generalization of findings to other populations. These limitations notwithstanding, the present study, to the best of our knowledge, is the first attempt to estimate suicidality among a large sample of university students in Ghana. The relatively large sample size enhances the generalizability of the findings to students in institutions of higher learning in Ghana. It prepares the grounds for future works to further study this underserved population and engender policies to address their mental health needs in Ghana.

## Conclusions

Findings from this study indicate that suicidal behaviours – ideations, plans, attempts, death wishes – are prevalent among university students in Ghana. The results also show that psychological distress and low self-esteem are predictors of suicidal ideation. Whereas psychological distress predicted suicide attempt, subjective wellbeing attenuated it. These findings underscore the need for the development of mental health services in this population in Ghana.
